# Six decades of malaria vector control in southern Africa: a review of the entomological evidence-base

**DOI:** 10.1186/s12936-022-04292-6

**Published:** 2022-10-02

**Authors:** Theresia Estomih Nkya, Ulrike Fillinger, Onyango P. Sangoro, Rose Marubu, Emmanuel Chanda, Clifford Maina Mutero

**Affiliations:** 1 International Centre of Insect Physiology and Ecology, Nairobi, Kenya; 2 University of Dar es Salaam, Mbeya College of Health and Allied Sciences, Mbeya, Tanzania; 3 World Health Organization-Regional Office for Africa, Brazzaville, Republic of Congo; 4 School of Health Systems and Public Health, University of Pretoria, Pretoria, South Africa

**Keywords:** Malaria vectors, Vector control, Malaria elimination, Southern African region

## Abstract

**Background:**

Countries in the southern Africa region have set targets for malaria elimination between 2020 and 2030. Malaria vector control is among the key strategies being implemented to achieve this goal. This paper critically reviews published entomological research over the past six decades in three frontline malaria elimination countries namely, Botswana Eswatini and Namibia, and three second-line malaria elimination countries including Mozambique, Zambia, and Zimbabwe. The objective of the review is to assess the current knowledge and highlight gaps that need further research attention to strengthen evidence-based decision-making toward malaria elimination.

**Methods:**

Publications were searched on the PubMed engine using search terms: “(malaria vector control OR vector control OR malaria vector*) AND (Botswana OR Swaziland OR Eswatini OR Zambia OR Zimbabwe OR Mozambique)”. Opinions, perspectives, reports, commentaries, retrospective analysis on secondary data protocols, policy briefs, and reviews were excluded.

**Results:**

The search resulted in 718 publications with 145 eligible and included in this review for the six countries generated over six decades. The majority (139) were from three countries, namely Zambia (59) and Mozambique (48), and Zimbabwe (32) whilst scientific publications were relatively scanty from front-line malaria elimination countries, such as Namibia (2), Botswana (10) and Eswatini (4). Most of the research reported in the publications focused on vector bionomics generated mostly from Mozambique and Zambia, while information on insecticide resistance was mostly available from Mozambique. Extreme gaps were identified in reporting the impact of vector control interventions, both on vectors and disease outcomes. The literature is particularly scanty on important issues such as change of vector ecology over time and space, intervention costs, and uptake of control interventions as well as insecticide resistance.

**Conclusions:**

The review reveals a dearth of information about malaria vectors and their control, most noticeable among the frontline elimination countries: Namibia, Eswatini and Botswana. It is of paramount importance that malaria vector research capacity and routine entomological monitoring and evaluation are strengthened to enhance decision-making, considering changing vector bionomics and insecticide resistance, among other determinants of malaria vector control.

## Background

The World Health Organization (WHO) together with the Roll Back Malaria (RBM) partnership have set targets for malaria elimination which are highlighted in the Global Technical Strategy for Malaria 2016–2030 [1]. In the southern Africa region (SAR) the elimination agenda is being pursued through initiatives such as Elimination 8 (E-8), launched in 2009 by the Southern African Development Community (SADC) [2]. The E-8 goal was to enable and accelerate the achievement of zero local transmission in the four frontline countries, Namibia, South Africa, Eswatini and Botswana, by 2020, and the second line countries, Angola, Zambia, Mozambique, and Zimbabwe, by 2030 through the provision of a joint platform for collaboration and joint strategic programming [3]. The goal of elimination in the SAR was premised on the documented reduction of malaria cases over the past 2000 to 2010 decade, attributed to improved case management and vector control, primarily indoor residual spraying (IRS) [4]. IRS with dichlorodiphenyltrichloroethane (DDT) played a significant role in the drastic reduction of morbidity and mortality in the region, going as far back as the 1940s in South Africa, Zimbabwe and Eswatini (formerly Swaziland) [5]. Before the introduction of IRS, malaria was hyper-endemic with intense seasonal transmission in endemic areas within the SAR [6–11]. The introduction of IRS was guided by surveys published between 1931 and 1957, reporting the wide spread of malaria vectors of the *Anopheles gambiae* complex and *Anopheles funestus* [6, 10, 12–15]. The commencement of IRS in the SAR dates to 1931 in South Africa when pyrethrum was tested on an experimental basis. From the mid-1940s DDT was used operationally targeting all malarious areas [4].

Successful implementation of IRS requires well-structured programmes, and countries like Eswatini and South Africa have National Malaria Control Programs (NMCP) dating back for almost seven decades [4, 16]. The upscaling of IRS with DDT was gradual [13, 17]. In Zimbabwe, DDT was introduced as a pilot in 1945 and evolved into a large-scale ‘barrier’ spraying programme to prevent epidemics and limit the spread of the disease to malaria-free areas [6]. After 1980 IRS applications expanded to reduce morbidity and mortality rather than only preventing epidemics [9]. In Botswana, even though the NMCP was established in 1974, IRS had been reported as far back as the mid-1940s [14], and by the 1950s, IRS with DDT became the main vector control method in the country [18]. The National Vector-Borne Diseases Control Programme (NVBDCP) of Namibia was established in 1991, however, malaria control efforts using IRS with DDT had been implemented since 1965 in selected areas and upscaled to full coverage in the 1970s [19]. Zambia established a comprehensive National Malaria Control Centre (NMCC) in the 1990s (1997) [20, 21] though the malaria control efforts started with the Mosquito Extermination Act of 1944 which mandated household management of outdoor water containers to eliminate mosquito breeding sites [21]. In the late 1950s, Zambia introduced IRS with DDT whereby municipal councils and the ministry of health sprayed urban areas, and mining companies administered and financed the program in mining districts [21]. Unlike the other SAR countries, Mozambique’s malaria control efforts collapsed due to the civil war in the late 1970s [22], after the country had been implementing IRS with DDT in selected southern parts of the country between 1960 and 1969 [23], as an upscale from the first round of implementation in 1946 [15, 24].

From the late 1980s onwards, countries started to shift at varying degrees from DDT to other insecticides belonging to the pyrethroid and carbamate groups in part due to the international lobby against persistent organic pollutants [25]. One of the notable successes in malaria control in the SAR was the Lubombo Spatial Development Initiative (LSDI) a tri-country project between South Africa, Eswatini (Swaziland) and Mozambique established in 2000, with one of its aims to decrease the transmission of malaria in the region largely using IRS in the at-risk regions and border areas [26, 27]. The success of the LSDI was evidenced in the substantial decreases in disease burden observed over 12 years across the three participating countries [26]. Control of malaria vector mosquitoes through insecticide-based IRS with insecticides, is still the primary malaria prevention tool in all malarious regions of SAR, nearly 80 years after its first use. Despite rigorous efforts, the ambitious control and elimination plans for the SAR countries [2, 3] have not been achieved [28, 29], with indigenous malaria cases increasing in several countries in recent years [3, 28].

A multitude of entomological studies from other (non-SAR) malaria-endemic regions in sub-Saharan Africa highlight the changing environmental conditions over the past decades, including land use and climate changes, changing vector dynamics and behaviours, and increasing insecticide resistance and more mobile human populations [30–32]. These studies highlight the importance of rigorous vector monitoring programs going hand in hand with vector control efforts to tailor evidence-based interventions. Here we review the literature for the six SAR countries over the past six decades with the aim to summarize the current knowledge base generated from entomological research and highlight gaps requiring attention for a more informed malaria elimination strategy.

## Materials and methods

Articles for this review were identified through a search on PubMed on 17th March 2021. The following search terms were used: “(malaria vector control OR vector control OR malaria vector*) AND (Botswana OR Swaziland OR Eswatini OR Zambia OR Zimbabwe OR Mozambique)”. The following inclusion criteria were applied for the review: (i) articles reporting data from Botswana, Eswatini (Swaziland), Zambia, Zimbabwe, and Mozambique; (ii) articles that included data on the following outcomes of interest: vector ecology, vector biology, vector control, long-lasting insecticidal nets, indoor residual spraying, insecticide-treated bed nets, larvicides, larval source management, habitat modification, habitat manipulation, biological control, house screening, house modification and mosquito repellents, the impact of vector control interventions, evaluation of vector control interventions, insecticide resistance and susceptibility, knowledge on malaria disease and malaria vectors; and (iii) articles reporting retrospective data that has not been published before. This review excluded studies that were opinions, perspectives, commentaries, retrospective analysis on secondary data already published in the original study, protocols, policy briefs, reports, and reviews.

This review excluded South Africa within the SAR region, as the country has well-established health research organizations and significant entomological expertise and training programs in stark contrast to the reviewed countries. To illustrate this, the same search terms were applied for South Africa only and the overall number of publications contrasted in the result section.

## Data analysis

Data from the selected articles were extracted onto a data extraction form created in Microsoft Excel for descriptive analysis of information on key study aspects matching our inclusion criteria such as design, intervention, aim and outcome. Studies conducted in laboratory settings using laboratory colonized malaria vectors, or vectors originating from the field but with the most analysis done in the laboratory, were classified as “laboratory studies” whereas those conducted under simulated field conditions (experimental huts) with field-collected or laboratory-reared mosquitoes were categorized as “semi-field studies”. “Field studies” included research activities that took place in the natural setting, or data used in the study originated from natural settings (field-collected mosquitoes that were analysed or tested in the laboratory).

## Results

**Fig. 1:**
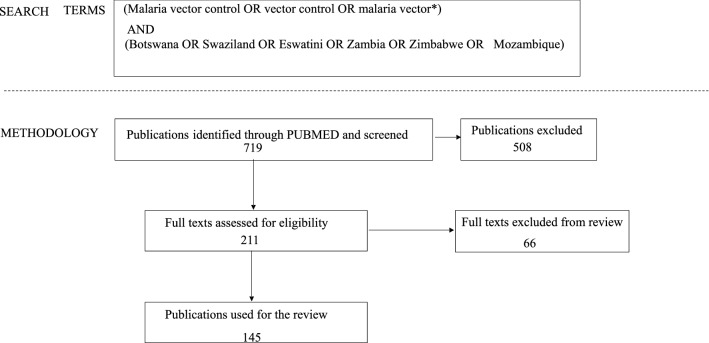
Search terms and methodology of selection of publications for review

The initial search yielded 719 articles. After screening titles and abstracts, we excluded 508 articles because they did not fulfil the inclusion criteria. Following a full-text review of 211 articles, a further 66 articles were excluded because they either reported on secondary data, were reviews, perspectives, or did not report on malaria vectors or control. This left only 145 articles published between 1963 and March 2021 for inclusion in this review, where in some cases one publication reported data from more than one of the target countries (Fig. 1).

## Publications by country

**Fig. 2:**
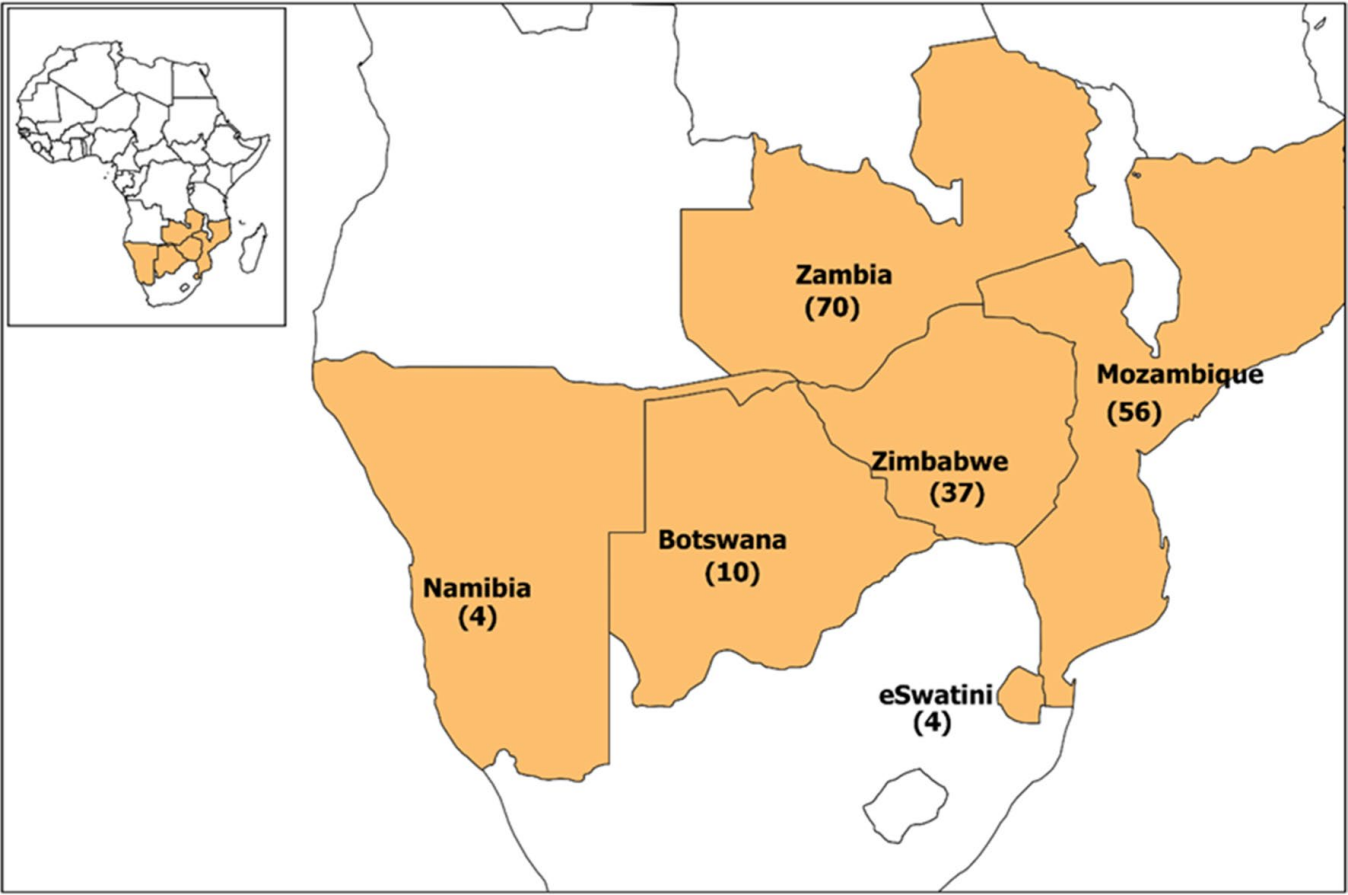
Reviewed publications by country. For publications involving multi-country studies, each country was tallied for the study separately. Frontline countries had set the target to eliminate malaria by 2020, second-line countries had set the target to eliminate malaria by 2030 [3]

**Fig. 3:**
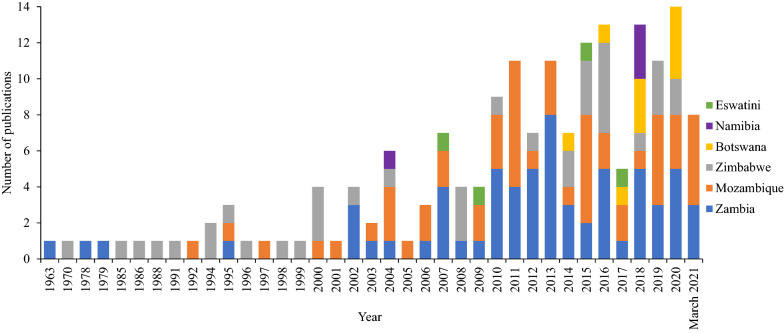
Number of research studies by year and by country. For studies involving multi-country studies, each country was tallied for publication separately

Of the 145 eligible studies generated over six decades, the vast majority (139) were implemented in three countries, namely Zambia (59) and Mozambique (48), Zimbabwe (32) whilst scientific publications from front-line countries for elimination, such as Namibia (2), Botswana (11) and Eswatini (4), were scant (Fig. 2). Furthermore, the research studies were aggregated by year and by country (Fig. 3). To put these findings in perspective, the same PubMed search with the same search terms were applied to South Africa over the same time frame from 1963 to 2021. This yielded 2134 results without applying any eligibility criteria, over three times more publications, compared to the 718 publications for the six SAR countries of interest in this review. For malaria-endemic countries in East Africa (Tanzania, Kenya, Uganda, Burundi, and Rwanda) the same search yielded 3085 publications with Tanzania accounting for over a third of them (1081).

To review the publications in the context of the malaria control efforts at the time, publications were aggregated into blocks as follows: malaria-endemic phase (1963–1999), scaling up of malaria control interventions (2000–2006), malaria decline phase (2007–2012), malaria plateau phase (2013–2015) and malaria resurgence phase (2016–2021) [3] (Table 1). Furthermore, the published research was grouped into two main thematic areas: malaria vector ecology and malaria vector control.

**Table 1:** Summary of research studies references from the reviewed SAR countries

Malaria elimination status	Country	Malaria endemic phase (1963–1999)	Scaling up of malaria control interventions (2000–2006)	Malaria cases decline phase (2007–2012)	Malaria cases plateau phase (2013–2015)	Malaria resurgence phase (2016–2021)
Frontline countries	Botswana (11)	0	0		[33]	[34–43]
	Eswatini (4)	0	0	[27, 44]	[45]	[46]
	Namibia (2)	0	0			[38, 47]
Second line countries	Mozambique (48)	[48–50]	[51–55]	[27, 56–68]	[69–78]	[36, 79–91]
	Zambia (59)	[92–95]	[96–99]	[100–114]	[115–128]	[84, 87, 129–148]
	Zimbabwe (32)	[149–158]	[159–164]	[165–168]	[121, 127, 169, 170]	[34, 84, 87, 139, 171–175]

For studies involving multi-country studies, each country was tallied for publication separately

Numbers in parentheses () are the total number of references for each category; numbers in brackets correspond to the reference citation

## Vector biology and ecology

### Species composition

This review explored the published evidence for vector incrimination, which is a prerequisite for understanding the role of anophelines in malaria transmission and has been used to determine which species are the most important vectors [176, 177]. There was no entomological evidence from Namibia or Eswatini. The incrimination of the primary malaria vector *Anopheles arabiensis* in Botswana is based on a small number of publications with limited spatial scale [35, 39, 42, 43]. *Anopheles gambiae* s.s. and *Anopheles funestus* have been identified as primary vectors in Mozambique [27, 51, 54, 66, 68, 70, 71, 81], Zimbabwe [121, 150, 151, 164, 171], and Zambia [102, 111, 113, 121, 126, 134, 140] (Table 2). The understanding of the local vector system is an essential step toward implementing effective vector control. The available studies on species composition in the SAR countries are limited in space and time, as there is inadequate routine malaria vector surveillance. Longitudinal changes in vector composition are inevitable as highlighted by recent reports of the re-emergence of *An. funestus* populations [87] and the discovery of a relatively diverse *Anopheles* fauna in Botswana [35]. This calls for caution and underscores the need for routine entomological surveillance to better understand the local vector ecology. Changes in the composition of local malaria vectors were also observed in Mozambique, where in the 1990s, both *An. funestus* and *An. arabiensis* were equally important vectors [50, 51] while in recent years *An. funestus* [54, 66, 68, 71, 81] seem to dominate, accounting for over 70% of all collected mosquitoes [54, 66].

**Table 2:** Research studies reference reporting on malaria vector identification and/or behaviour in the review countries of the SAR

Malaria elimination status	Country	Malaria endemic phase (1963–1999)	Scaling up of malaria control interventions (2000–2006)	Malaria cases decline phase (2007–2012)	Malaria cases plateau phase (2013–2015)	Malaria resurgence phase (2016–2021)
Frontline countries	Botswana (6)	0	0	0	[33]	[33, 35, 39, 40, 42, 43]
	Eswatini (0)	0	0	0	[45]	0
	Namibia (0)	0	0	0	0	0
Second-line countries	Mozambique (21)	[48, 50]	[51, 52, 54, 56, 208]	[27, 58, 60, 62, 64, 66, 68]	[55, 70, 71, 74]	[36, 81, 83]
	Zambia (23)	[93, 150]	[98, 99, 164]	[101, 102, 105, 108–113]	[121, 123, 126]	[129, 130, 134, 140, 143, 171]
	Zimbabwe (10)	[150, 151]	[159, 164]	[165]	[121, 169, 170],	[171, 172]

For studies involving multi-country studies, each country was tallied for publication separately

Numbers in parentheses are the total number of references for each category; numbers in brackets correspond to the reference citation

Numerous factors can contribute to vector population change such as vector control interventions (LLINs, IRS), human behaviour and climate change [176–178]. The effect of vector control intervention on species composition was observed in Mozambique after scaling up of IRS and LLINs [72, 74] and in Zimbabwe where suppression of *An. gambiae* s.s. by indoor spraying [150] was observed which could be explained based on the vector’s endophilic tendency of feeding and resting indoors [179] which is the main target of IRS. On the other hand, it has been shown that the selected sampling strategy can significantly affect what vector species are collected and reported, hence routine entomological surveillance must choose the sampling strategies such that all vectors can be identified. The literature for the reviewed countries is highly diverse with sampling in Mozambique done with CDC light traps, resting catches, exit collections, man baited double net, and knockdown collections [54, 70, 81] while in Zimbabwe the primary collection methods were man-baited nets both indoors and outdoors, complemented by pit traps [150, 171, 172]. The importance of the sampling methods on specie composition is also highlighted by a study from Zambia, where window exit trap collections were dominated by *An. arabiensis* despite a strong presence of *An. funestus* and *An. gambiae* s.s. in the study area [111].

### Larval ecology

Few larval ecology studies of these vectors in Botswana [33, 42], Zambia [105, 112], and Eswatini [45] are limited in scope and cannot inform potential intervention strategies such as larval source management (Table 2). A novel approach to mapping larval breeding sites has been used in Eswatini [45] which might have the potential to predict larval habitats in remote areas and inform interventions if more thoroughly supported by on-the-ground entomological surveys. The remotely sensed data seems to suggest that permanent habitats associated with farming play a major role in malaria vector production in Eswatini. A similar remote sensing approach was used in a small study area in southern Zambia which is characterized by seasonal streams [105]. Again, permanent to semi-permanent habitats were signified as the major breeding sites of the local vectors. However, there is a need to expand such surveys in space and time in the target countries to be able to arrive at more generalizable results across different eco-epidemiological settings for example to inform the suitability of larval source management in a selected hotspot or elimination areas. The *An. funestus* larval ecology is generally not well studied in sub-Saharan Africa due to its affiliation with swampy, permanent, and difficult to access water bodies [180] and hence, it is not surprising that no information is available from the reviewed countries that have identified this species as the main malaria vector.

### Adult vector ecology

Knowledge of vector resting behaviour, host-seeking, biting, host preference and vector competence is essential for the strategic implementation of vector control. For instance, knowledge of vector resting behaviour is essential if that behaviour is the target for control, such as the use of IRS. LLINs aim to control vectors that feed indoors when people are asleep. House entry and indoor resting habits of vectors are hence essential for this tool to be effective [181, 182]. Investigating host-seeking behaviour, host preferences and the presence of sporozoites in *Anopheles* mosquitoes help gauge their role as vectors of malaria [171]. Mosquito biting behaviour includes biting seasonality, diel biting activity (i.e., peak biting in a 24-h cycle) and preferred biting location relative to a house (indoor vs outdoor) [182]. IRS, which is the core intervention in the frontline countries for malaria elimination requires vectors to rest indoors either before or after biting a human. Yet there is a lack of published data on vector resting behaviour from Eswatini and Namibia, and only two small studies from Botswana, [35, 43].

Host preferences and times of biting of local malaria vectors have an impact on the disease transmission and vector control tools need to be aligned with the vector’s behaviour to interrupt man-vector contact [183]. For instance, LLINs and IRS that target mostly indoor feeding and resting mosquitoes are not efficient to control vectors that feed and rest outdoors leading to residual malaria transmission [184]. *An. arabiensis* has been reported to contribute to residual malaria transmission due to its tendency to feed outdoors [185], however, in the reviewed countries, three studies from Botswana, Mozambique and Zambia exclusively reported this specie to feed and rest indoors [39, 51, 113]. These observations could be explained by the fact that most mosquitoes were collected indoors. However, this differs from other studies reported elsewhere in sub-Saharan Africa, where *An. arabiensis* tends to feed and exit domiciles [186, 187]. Further studies are needed to establish this vector’s feeding and resting behaviour in the SAR, to determine its role in the residual transmission and its ability to avoid IRS, which can impact the elimination goal.

LLINs target to interrupt contact between humans and vectors as they form a physical and chemical barrier against mosquitoes. When mosquitoes try to bite, they are not only blocked by the netting but also killed by the insecticide coating. LLINs protect humans while sleeping and the peak feeding time will inform the impact of the intervention. Data from the reviewed countries are largely out of date, dating back a decade or longer. These available reports show clear late-night indoor biting peaks. *An. funestus* had varying peak hours of feeding between 2100 and 2400 h in Mozambique [51] and 2200–2300 h and 0200–0400 in Zimbabwe [150] and in Zambia between 2400 and 0600 h [113]. However, biting peaks for *An. funestus* have been shown to differ elsewhere in Africa [188–191]. These observations of indoor resting after feeding for *An. gambiae* s.s. and *An. funestus* s.s. seems to confer with the known resting behaviour of these species [181, 182]. There is a need for updated information given that high vector control pressure selects for avoidance behaviours.

Studies on vector parameters such as seasonality and vector competence are scarce in these reviewed countries. *An. funestus* was reported to be abundant during all seasons in Mozambique [54]. In Zimbabwe, vector peak populations were observed in March and *An. funestus* densities were higher in the wet than the dry season [150]. Expectedly, the vector peak density is aligned with the rainy seasons in these countries as demonstrated elsewhere [192–194]. In Zimbabwe, it was observed that in unfed *An. funestus* group preferred seeking a human host [182]. The Human Blood Index (HBI) represents the proportion of blood meals derived from humans by mosquito vectors, whereas in Zimbabwe, 64% of collected *An. funestus* fed on humans and had a *Plasmodium falciparum* infection rate of 1.8% [171]. The few studies on vector competence from SAR observed the man survival rate for *An. funestus* and *An. arabiensis* was 79% with varying sporozoite rates, in Mozambique [51]. A commonly used measure of malaria transmission intensity is the entomological inoculation rate (EIR), defined as the product of the human biting rate (HBR) and sporozoite infection rate (SIR). This review noted a dearth of information on this parameter, with reports from Zambia on EIRs of *An. funestus* s.s. 39.6 and *An. gambiae* s.s. of 5.9 [134].

### Secondary vectors

More recent studies highlight the role of secondary malaria vectors such as *Anopheles merus* from Mozambique [58], *Anopheles coustani* s.l. and *Anopheles squamosus* from Zambia [109], as potential local malaria vectors. Although generally believed to be of negligible importance, *P. falciparum* sporozoites were detected in numerous *An. squamosus* specimens, in Botswana and *Anopheles parensis* were found to have human blood [35]. These findings suggest that indoor vector control strategies might not be sufficient for the elimination of malaria in this region. As more studies are implemented in recent times, the complexity of malaria transmission becomes apparent. Implementing vector control independent from entomological evidence might in part be responsible for the persistence of malaria in this region.

## Vector control

Vector control has a long history in the reviewed countries with IRS and to a lower extent LLINs yet there is surprisingly little research data available on the feasibility and impact of interventions.

### Indoor residual spraying (IRS) and long-lasting insecticide-treated nets (LLINs)

Despite extensive use, the effectiveness, efficacy, cost, and acceptance of IRS have not been assessed in the frontline countries for malaria elimination. Publications from second-line countries for elimination Zambia [96, 104, 114, 116, 117, 119, 124, 132, 138, 141, 145, 148], Mozambique [27, 53, 56, 59, 62, 63, 85, 89, 90] and Zimbabwe [151, 152, 157, 159, 175] are more abundant, though still comparatively few. The evaluation of different classes of insecticides for IRS has shown that pyrethroids, organochlorides, carbamates and organophosphates have different degrees of effectiveness and efficiency [195]. The organochloride DDT significantly contributed to controlling the spread of malaria in the SAR, but in part due to the environmental concern of this chemical [196, 197], other insecticides were introduced for malaria control.

While the impact of bed nets has been extensively tested and proven in sub-Sahara Africa few reports on effectiveness, efficacy, cost, accessibility, and ownership of bed nets exist from the reviewed countries. As observed with other topics, second-line elimination countries such as Mozambique [57, 61, 75, 79, 80, 89], Zambia [97, 100, 107, 114, 119, 120, 124, 132, 133, 139] and Zimbabwe [139, 162, 166, 168, 175] have more data than elimination countries which do not place a strong emphasis on LLINs in their national malaria control programs. Current LLINs are manufactured for durability. However, research should periodically confirm the field performance of bed nets and barriers to use. Very few studies from Zambia [128] and Mozambique [77, 78, 88] have observed the durability and integrity of LLINs.

The combined benefits of IRS and LLINs on malaria are of high interest to policy. In Mozambique, no substantial difference was detected in the overall reduction of malaria cases between districts implementing IRS and LLINs [114]. However, this trend is not uniform in the SAR, as Zambia has reported a continuous high burden of malaria after 7 years of implementation of IRS and LLINs in Luapula province [124]. Evidence that incremental impact is achieved when combining IRS and LLINs remains limited and inconsistent. In Zambia, in fourteen population clusters of approximately 1000 residents each in Luangwa and Nyimba districts, where universal coverage targets for LLIN utilization have been achieved, supplementing LLINs with IRS using pyrethroids reduces malaria transmission below levels achieved by LLIN use alone [132]. Concerning insecticide resistance that may occur from selection pressure in such a combination, the authors further recommended supplementing LLINs with IRS using non-pyrethroid insecticide classes, and in addition, attaining far greater transmission reduction [132].

### Larval source management

Larval source management refers to the targeted management of mosquito larval habitats, to suppress mosquito larval and pupal abundance. Techniques used in LSM include environmental management and manipulation, larviciding, biological control or combinations of these methods [198]. In SAR, the use of LSM has not been widely studied nor used. However, there is some documented evidence of the success of LSM in reducing malaria incidence in the pre-era of IRS with DDT, such as seen in the Zambian copper mines, whereby environment management was used to destroy the larval stages of mosquitoes [199]. Two experimental studies in Botswana demonstrated a reduction of larval densities when the biological larvicide *Bacillus thuringiensis serovar israelensis* (Bti) was applied, however, these were not trials assessing the impact of the tool on malaria transmission [34, 37].

The effectiveness and efficacy of LSM using biological larviciding have been demonstrated elsewhere in Africa [200–207], however, more data is needed to demonstrate the impact of this tool in the SAR in the context of malaria elimination.

### Repellents

Repellents may provide a personal protection solution during outdoor activities [181]. In SAR, there is a paucity of research studies on repellents for personal or space protection. The very few studies available are spaced over time and are of small scale within controlled conditions ranging from mosquito coils to spatial repellents [95, 136, 166]. In the recent decade, spatial repellents are being assessed for integrated vector management, but limitations exist in the residual effect of the repellent and the need for external power or heat for the diffusion of the volatiles [136].

### Intervention acceptance and uptake

Community acceptance, ownership, and perceived effectiveness of vector control tools have been rarely conducted in the reviewed countries (Mozambique [59, 85], Zambia [57, 97]) In Zambia, despite the members of the community knowing that bed nets are useful in reducing the frequency of getting malaria, very few of those questioned owned a bed net [97]. Thus, such studies could be a starting point for the further expansion of an integrated approach to vector control in these countries.

### Insecticides and resistance

Insecticides used or tested for malaria control in SAR include pyrethroids, carbamates and organochlorides. These insecticides have been used for IRS and in LLINs. Despite a high reliance on insecticides for the control of malaria vectors for decades, insecticide resistance is not well documented, with few reports from Mozambique [55, 56, 60, 62, 64, 65, 74, 83, 90, 208], Zimbabwe [121, 165, 169, 170] and Zambia [110, 111, 121, 122, 143], one from Botswana [40]. Information from Eswatini and Namibia is lacking altogether. For effective management and control of insecticide resistance in malaria vectors, frequent detection and monitoring of vector susceptibility and associated resistance mechanisms are crucial [209]. *An. funestus* has been reported to be resistant to pyrethroids [60, 62, 64, 111, 121, 143, 208], and the carbamate bendiocarb [170]. Low-level resistance to the carbamate propoxur in *An. arabiensis* has been reported in Mozambique [55], while resistance to DDT and the pyrethroids was detected in *An. gambiae* s.s. [111, 122]. Resistance mechanisms are largely unknown, with one report on the genotypic presence of target site mutation (knockdown resistance) [39] and a few on metabolic resistance associated with elevated p450 monooxygenase activity and *acetylcholinesterase* levels [60, 143, 208].

## Discussion

This review chronicles the vector research and control activities in six countries of the SAR for almost six decades starting from 1963 to 2021. Much of the literature was generated during the past two decades (2000–2021). There has been very little entomological research in the reviewed SAR countries before 2000, with just one publication per year in Mozambique, Zimbabwe, or Zambia. As the SAR had set goals for malaria elimination and in some countries such as Eswatini, Botswana and Namibia failing to meet their elimination targets of 2020, it will be necessary for these countries to update the knowledgebase of vector research and control by conducting routine entomological (vector) surveillance and evaluation. The historical literature generated may not hold as environmental landscapes continue to change.

Within the context of malaria elimination, the value of understanding the distribution and ecology of local malaria vectors and their ability to sustain malaria transmission cannot be understated. This review found that literature on malaria vector behaviour and control in the reviewed regions is limited, out of date or non-existing at all as for Namibia and Eswatini. However, the studies identified and reviewed herein provide a useful starting point for identifying the gaps and setting goals for future research areas.

SAR is large in a geographical context and extremely heterogeneous and fragmented with countries having distinct environments with a highly marked relief. The geographic landscape and topology differ from one country to the next and some even differ from one micro-district to the next within a country. Furthermore, the climate includes a range from semi-arid savanna to tropic with extreme differences in rainfall. For instance, the characterization of breeding sites differed for the vector *An. arabiensis* in Botswana [42, 43] and Zambia [112], thus highlighting the importance of local knowledge for proper planning of vector control activities such as larval source management. The human environment is equally varied, from the housing structures, human movement across borders, economic activities, traditions, and socio-demographics. This heterogeneity complicates the control and elimination of malaria in the region, especially where cross-border transmission is important.

The scarcity of ecological vector research data hampers the potential exploration of novel intervention strategies, such as genetic control tools. The few studies investigating vector bionomics in the region, seem to suggest that there are more vectors involved in malaria transmission, than the primary malaria vectors *An. funestus*, *An. arabiensis* and *An. gambiae*. The absence of data from Namibia and Eswatini in this context is therefore even more surprising. Overall, publications were found to be very limited in scale, usually associated with the presence of research institutions in a few selected areas. This eco-epidemiological bias poses the question of whether the local vector profile is representative of the country. While it is generally thought that *An. arabiensis* tends toward exophilic and exophagic [210], results reported in this review demonstrated a regional difference in behaviour. These results suggest entomological efforts to reduce transmission could benefit from different vector control approaches appropriate for the local situation.

A major challenge for improved vector control in the region is the selection and combination of appropriate strategies that will efficiently provide the maximum impact for malaria elimination. In the reviewed countries, the impact of IRS to reduce malaria transmission has not been meticulously monitored, so it is difficult to unequivocally state the extent of the impact and the potential gaps. The current evidence is limited to a few localities [96, 114], however, more robust studies are needed. It is important to note that acceptance and coverage of IRS achieved during earlier vector control campaigns may not be reproducible due to community fatigue and change to other social demographics like an increase in income leading to the building of more modern houses thus refusing IRS. However, national coverage may not be required for the elimination and focal application of IRS to malaria-endemic areas or reporting epidemics may be achievable. It is however likely, that impact would benefit from combinations of tools and the addition of strategies that are less dependent on the currently used insecticides.

The impact and effectiveness of LLINs have been demonstrated in sub-Sahara Africa [211]. There is potentially room to increase coverage of LLINs as IRS is only used for targeted hotspots. Like IRS, to achieve malaria elimination in SAR, focal use of LLINs may be a good approach. New insecticides are being introduced to be used in IRS, such as neonicotinoid Clothianidin, however, they have not been evaluated in the SAR, thus the need to explore these new molecules in the context of tackling insecticide resistance and achieving malaria elimination.

The lack or rather scarcity of entomological information in the SAR stems from a lack of resources, both human and infrastructure. There is a need to improve SAR entomological surveillance capacity within countries and across the border as this region is interconnected via many aspects. The paucity of data in Eswatini, Namibia and Botswana could be a result of the absence of local capacity in both human and infrastructure to conduct entomological surveys. The need to build entomological capacity at the national level requires significant financial and technical investment. However, vector control interventions will have the greatest impact when implemented based on real-time data. As SAR makes progress in eliminating malaria, it will be challenging to measure the impact as fewer cases will be detected. Serological approaches that measure malaria and mosquito exposure might be useful additional surveillance methods.

Whether widespread or focal insecticide-based interventions are used, malaria vector populations will have to be monitored for insecticide resistance. The existing data is insufficient for some of the reviewed countries. There is an urgent need to establish national insecticide resistance detection and monitoring plans, which are crucial for maintaining the efficacy of the current tool.

For sustainability of the currently implemented strategies (IRS and LLINs), there is a need to assess the cost-effectiveness as well as the timely deployment. One of the setbacks in IRS implementation in the SAR is logistics involving delays in shipments thus receiving insecticides when the spraying season is over or when the insecticides have expired [195, 212]. The financial and technical cost needs a thorough assessment to drive malaria transmission down to zero while these approaches should be judiciously considered and supported by both government and donors to achieve the goal of malaria elimination.

New vector control approaches [213] and new strategies [31] are gaining more interest; therefore, consideration should be made for these interventions. Some activities that may have an immediate impact on SAR include (1) house screening to prevent mosquito entry into homes [214, 215]; (2) LSM using biological larvicide targeting immature stages of mosquitoes [216]; and (3) the re-emerging strategy The Global vector control response 2017–2030 (GVCR) emphasises increased capacity, improved surveillance, better coordination and integrated action across sectors and diseases [31].

This review had some limitations. The review focused on peer-reviewed publications in the PubMed search engine, thus most of the entomological surveillance reports that are on national malaria programmes of these countries were excluded because they were not published. The exclusion of these reports was on the scientific merits of data in peer-reviewed publications. Whilst not peer-reviewed these documents such as annual reports and strategic documents could have provided information on routine surveillance or operational research in entomology if any available, that could support some of the programmatic decisions such as their choice of insecticide for their IRS campaigns.

## Conclusions

The review aimed to assess the current knowledge and highlight gaps that need further research attention to strengthen evidence-based decision-making toward malaria elimination. The reviewed data was derived from peer-reviewed publications mostly from the second-line countries for malaria elimination as opposed to the first-line countries for malaria elimination. The review reveals a dearth of information about malaria vectors and their control, most noticeable among the frontline elimination countries: Namibia, Eswatini and Botswana. It is of paramount importance that malaria vector research capacity and routine entomological monitoring and evaluation are strengthened to enhance decision-making, considering changing vector bionomics and insecticide resistance, among other determinants of malaria vector control. The paucity of data for Namibia and Eswatini calls for exploration of contributing factors thus calling for further reviews on local capacity in both human and infrastructure. Entomological surveillance provides the foundation necessary to optimize vector control strategies and is crucial for the malaria elimination success in SAR.

## Data Availability

Information was searched and obtained from published data in the PubMed search engine. The datasets used and/or analysed during this study can be obtained from the corresponding author on reasonable request.

## Abbreviations

IRS: Indoor residual spraying
IVM: Integrated vector management
LLINs: Long-lasting Insecticidal Nets
LSM: Larval source management
SADC: Southern African Development Community
SAR: Southern Africa Region
WHO: World Health Organization
